# Reference Equation of a New Incremental Step Test to Assess Exercise Capacity in the Portuguese Adult Population

**DOI:** 10.3390/jcm12010271

**Published:** 2022-12-29

**Authors:** Rui Vilarinho, Ana Toledo, Carla Silva, Fábio Melo, Leila Tomaz, Luana Martins, Tânia Gonçalves, Cristina Melo, Cátia Caneiras, António Mesquita Montes

**Affiliations:** 1 FP-I3ID, Escola Superior de Saúde-Fernando Pessoa, 4200-253 Porto, Portugal; 2 Center for Rehabilitation Research (CIR), School of Health, Polytechnic Institute of Porto, 4200-072 Porto, Portugal; 3 Healthcare Department, Nippon Gases Portugal, 4470-177 Maia, Portugal; 4 Microbiology Research Laboratory on Environmental Health (EnviHealthMicroLab), Institute of Environmental Health (ISAMB), Faculty of Medicine, Universidade de Lisboa (ULisboa), 1649-028 Lisbon, Portugal; 5 Multidisciplinary Research Center of Egas Moniz (CiiEM), Egas Moniz School of Health and Science, 2829-511 Almada, Portugal; 6 Institute for Preventive Medicine and Public Health, Faculty of Medicine, Universidade de Lisboa (ULisboa), 1649-028 Lisbon, Portugal; 7 Department of Physiotherapy, Santa Maria Health School, 4049-024 Porto, Portugal

**Keywords:** exercise tests, exercise tolerance, step mode testing, interpretability

## Abstract

Step tests are important in community- and home-based rehabilitation programs to assess patients’ exercise capacity. A new incremental step test was developed for this purpose, but its clinical interpretability is currently limited. This study aimed to establish a reference equation for this new incremental step test (IST) for the Portuguese adult population. A cross-sectional study was conducted on people without disabilities. Sociodemographic (age and sex), anthropometric (weight, height, and body mass index), smoking status, and physical activity (using the brief physical activity assessment tool) data were collected. Participants performed two repetitions of the IST and the best test was used to establish the reference equation with a forward stepwise multiple regression. An analysis comparing the results from the reference equation with the actual values was conducted with the Wilcoxon test. A total of 155 adult volunteers were recruited (60.6% female, 47.8 ± 19.7 years), and the reference equation was as follows: steps in IST = 475.52 − (4.68 × age years) + (30.5 × sex), where male = 1 and female = 0, and r^2^ = 60%. No significant differences were observed between the values performed and those obtained by the equation (*p* = 0.984). The established equation demonstrated that age and sex were the determinant variables for the variability of the results.

## 1. Introduction

The assessment of exercise capacity is a common component in preventive and rehabilitation programs to detect changes in physical function, especially in exercise tolerance function, and, consequently, to establish prognosis [1,2]. It also provides parameters for the prescription of exercise programs and response to intervention [3]. Cardiopulmonary exercise testing (CPET) is considered the gold standard method used to assess exercise capacity, by providing a measure of the maximum oxygen consumption (VO_2_max), which in turn is the gold standard measure for the assessment of cardiorespiratory fitness (CRF) [4]. Treadmills and cycle ergometers are indicated to assess CRF [1]; however, their use is not always feasible in all settings where rehabilitation programs can be implemented [5], especially in community- [6,7,8] and home-based [9,10,11] programs, because they have high costs and require both specialized instruments and trained personnel [1]. To overcome these limitations, field tests can be more affordable, simple to apply, and better related to patients’ demands during activities of daily living [2,12]. Because of this, step tests are a suitable alternative which, in addition to the advantages mentioned above, require little equipment (an easily transportable platform), and the stepping skill requires little practice [1]. Additionally, step tests with an incremental and externally paced profile can provide a maximal cardiorespiratory response [13].

Recently, a new incremental step test was developed for patients with COPD with promising results for their measurement properties (correlation values of 0.50 and 0.46 for construct validity and ICC = 0.96 for reliability) and its application proved to be feasible in the home environment because this data collection was performed at participants’ homes [14]. This test is composed of an incremental profile using a digital recording with a timed metronome step cadence through 15 levels of step cadence, each of a 1-min duration. The timed metronome sets the step cadence, which starts at 10 steps/minute and increases 2 steps/minute every 1 min, with a step cadence maximum of 38 steps/minute (level 15). According to these characteristics and the good results on their measurement properties in the COPD population, its general application for other clinical populations (e.g., other chronic respiratory diseases and cardiac diseases) seems to be recommended, along with the assessment of the respective measurement properties [15]. 

In addition to the measurement properties, the clinical interpretability of field tests is also important and transversal to all clinical populations, providing a clear interpretation of their performances through comparisons, for example, with reference equations generated from data of apparently healthy populations [16]. This also yields a definition of the utility of this new step test as an outcome measure of exercise capacity and assess the effectiveness of interventions [15]. Currently, the clinical interpretability of this new incremental step test is unknown. Therefore, the need to produce a country-specific reference equation based on its performance is important. This study aims to establish a reference equation of this new incremental step test (IST) to assess exercise capacity in the Portuguese adult population.

## 2. Materials and Methods

### 2.1. Study Design

This cross-sectional study was conducted between April 2021 and November 2022 in people without disabilities. Ethical approval for this study was obtained from the Ethics Committee of the School of Health—Polytechnic Institute of Porto (E0134, 13 April 2020). This study was also registered at ClinicalTrials.gov (registry number NCT04801979).

### 2.2. Participants

The study was conducted on people without disabilities in the north of Portugal. Each investigator responsible for the data collection advertised the study in their hometown and the surrounding areas. Interested participants contacted each investigator directly to participate in the study. According to another study in which it also determined reference equations for field tests for Portuguese adults [17], to achieve maximum representativeness from community-dwelling people, participants aged equal to or above 18 years and both sexes, with the most prevalent age-related conditions (e.g., hypercholesterolemia, hypertension, and diabetes) were included in the study [18]. The exclusion criteria were the presence of one or more of the following conditions: acute (within the past 4 weeks) or chronic respiratory disease; cardiac disease; signs of cognitive or neuromuscular impairment; and significant musculoskeletal disorder (e.g., ankylosing spondylitis) that could interfere with the ability to perform the step test. Subjects using walking aids were also excluded.

### 2.3. Data Collection

Sociodemographic (age and sex), anthropometric (weight, height, and body mass index [BMI]), clinical (comorbidities and smoking status, i.e., current smoker, past smoker, or never smoker), and physical activity (PA) data were collected. PA was assessed using the brief physical activity assessment tool [19]. This tool consists of two questions that consider the frequency and duration of moderate and vigorous PA during a usual week. Each question is rated in a 1–4 scale. Total scores vary from 0 to 8 and they yield further classification of the individual as “insufficiently active” (score 0–3) or “sufficiently active” (score ≥ 4) [17]. These classification categories showed good construct validity (0.394 ≤ ρ ≤ 0.435; 0.36 ≤ κ ≤ 0.64; 0.5 ≤ sensitivity ≤ 0.75, 0.74 ≤ specificity ≤ 0.91) in patients with various health conditions [19,20,21]. 

Age, sex, height, weight, BMI, smoking status, and PA were chosen as independent variables for the development of the reference equation due to their simple collection in clinical practice and have been included in previous reference equations for other field tests (e.g., 6-min walk test and incremental shuttle walk test) [16].

Participants performed two repetitions of the IST with a minimum rest period of 30-min between them. Data were collected by physiotherapy final year undergraduate students under the coordination of trained physiotherapists with experience in applying field tests to assess exercise capacity. The best test (highest number of steps) was used in the analysis. Due to the COVID-19 pandemic, all subjects participating in the study used a face mask. Although some of the available literature suggests that negative effects of using face masks during exercise in healthy individuals are negligible and unlikely to impact exercise tolerance significantly [22,23], some studies found a negative impact which results in decreased exercise performance [24,25]. However, the use of face masks was a factor expressed by most of participants in order to participate in data collection.

### 2.4. Incremental Step Test

The IST was designed to provide an incremental profile by using a digital recording with a timed metronome step cadence and a 20 cm tall platform (Max Aerobic step, Mambo, Tisselt, Belgium). The test consists of 15 levels, each of a 1-min duration. The timed metronome sets the step cadence, which starts at 10 steps/minute and increases 2 steps/minute every 1 min, with a step cadence maximum of 38 steps/minute (level 15). The maximum test duration is 15 min [14]. Heart rate (HR) and SpO_2_(%) were monitored during the tests with a pulse oximeter (PalmSAT 2500 Series, Nonin Medical, Minnesota, USA). The criteria to stop the test were as follows: unable to maintain the required step cadence for 10 s; requested by the participant; if abnormal physiological responses occurred (i.e., persistent peripheral oxygen saturation <85%); and reported symptoms of exertion intolerance (e.g., chest pain, intolerable dyspnea or leg fatigue, dizziness, vertigo, and pallor).

### 2.5. Sample size and Data Analysis

For this study, the sample size for multiple linear regression to establish the reference equation of the IST was determined according to Green’s (1991) recommendations [26]:

N > 50 + 8 m,(1)

where N is the total sample size and m is the number of independent variables. Because seven independent variables (age [numerical], sex, height, weight, BMI, smoking status, and PA) were considered, a minimum of 106 participants was necessary during the recruitment phase.

The statistical analysis was performed using SPSS version 27.0 (IBM Corporation, Armonk, NY, USA). The level of statistical significance was set at *p* < 0.05. The normality of data distribution was verified using the Kolmogorov–Smirnov or Shapiro–Wilk tests. Descriptive statistics were used to describe the samples, and the data are presented as mean ± standard deviation or median [percentile 25–75]. Comparisons between age decades were explored using the Kruskal–Wallis test with Bonferroni’s correction.

The development of the reference equation was performed using a random selection of 80% of the included participants. According to the analysis of data normality, Spearman’s correlation coefficients were calculated to explore the association between the dependent variable (number of steps) and the independent variables (age, sex, height, weight, BMI, smoking status, and PA). The strength of the correlations was classified according to British Medical Journal guidelines: significant correlation coefficients of 0–0.19 as very weak; 0.2–0.39 as weak; 0.4–0.59 as moderate; 0.6–0.79 as strong; and 0.8–1 as very strong [27]. The dependent variables that were significantly correlated with the independent variables were suited in a further selection stepwise multiple regression. The assumptions of the multiple regression were confirmed, namely the linear relationship between the dependent and independent variables, absence of multicollinearity within the independent variables, homoscedasticity, outliers, and normality of residuals) and r^2^ was used to assess the performance of the model. The validity of the reference equation created was further assessed with the remaining 20% of the sample and it consisted of comparing the results achieved and those predicted by the equation with the Wilcoxon signed-rank test.

## 3. Results

In total, 176 volunteers were recruited, and 14 participants were excluded due to the presence of a respiratory disease (*n* = 3), musculoskeletal disease (*n* = 6), status of long-COVID-19 (*n* = 4), and the use of walking aids (*n* = 1). From these 162 participants, 7 were excluded for presenting persistent high levels of BP before the performance of the step test. Therefore, 155 adults participated in the study (60.6% female; mean age, 47.8 ± 19.7 years; minimum age, 19 years; maximum age, 93 years). Most were non-smokers (63.2%), the mean of BMI was classified as normal (24.9 ± 3.6 kg/m^2^) [28], and the BMI minimum and maximum were 17 and 37 kg/m^2^, respectively. According to PA scores, 85 participants (54.8%) were classified as insufficiently active and the others 70 participants (45.2%) were classified as sufficiently active (Table 1).

### 3.1. Performances on the Incremental Step Test by Age Decade

Participants (*n* = 155) performed a mean of 328 [170; 360] steps in the IST. Table 2 shows the median values of performance for the IST by age decade. Performance in the IST was found to decrease with age (*p* < 0.001) (Table 2).

### 3.2. Reference Equation

According to 80% of the sample (*n* = 124) and based on the best performance in the IST, the reference equation was developed. There were significant correlations between the number of steps in the IST and age (r = −0.68, strong correlation, *p* < 0.001), sex (r = 0.11, very weak correlation, *p* = 0.04), and height (r = 0,33, weak correlation, *p* < 0.001), but not weight, smoking status, BMI, and PA (*p* > 0.05). The model of the stepwise multiple regression showed that age and sex explained 60% (*p* < 0.001) of variability in the IST (Table 3). The reference equation for the number of steps was:

Number of steps (IST) = 475.52 − (4.68 × age years) + (30.5 × sex), where male = 1 and female = 0.(2)

### 3.3. Validity of the Reference Equation

According to 20% of the sample (*n* = 31), no significant differences (*p* = 0.984) were observed between the actual values performed by participants (319 [141; 360] steps) and those obtained by the equation (290 [173; 339] steps).

## 4. Discussion

This study determined a reference equation for IST performance and showed that the variability of results was explained by sex and age. It is important to mention that these values were obtained from participants over a wide range of ages between 19 and 93 years. The inclusion of age as a determinant variable for the variability of results was expected because the aging process causes skeletal muscle contractile function loss [29] and lower oxygen consumption [30], leading to a worse exercise capacity. Another explanation for these results is the greater probability of the difficulty for older adults to negotiate stairs as a marker of functional decline, which can influence the performance in step mode testing. This difficulty is not only associated with reduced lower-limb strength, but also with reduced sensation and balance, and an increased fear of falling [31]. In fact, age has been the most indicated predictor of performance in exercise field tests, namely in other steps tests [32,33,34], walking tests [16,17,35], and upper-limb exercise tests [17].

We also expected that sex could influence the performance of the IST. Sex is considered a strong predictor of exercise capacity [36] that is consistently observed in reference equations for the prediction of exercise capacity in field tests, especially in walking tests [35,37,38] and step tests [32,33,34]. 

On the other hand, we expected that other independent variables could influence the performance of the IST, highlighting body composition. Body composition, through anthropometric measures, can be a determinant variable in the assessment of exercise capacity [39]; however, the variables used in our study (weight, height, and BMI) did not influence performance in the IST. We hypothesized that weight could be a predictor of a smaller number of steps performed. Overweight, associated with more fat accumulation, increases the workload on horizontal (walking) and vertical displacements, which normally occur during step mode testing [40,41,42]. Despite the controversy over whether BMI is the best measure of obesity, BMI ranges are still based on excess body fat [28]. As such, it was expected that this variable also could negatively influence performance in terms of the number of step tests; however, this was not observed in our reference equation. The lack of variability in BMI values in our sample was not a limitation for this observation because the participants included in the development of the reference equation (80% of participants) presented a wide variety of BMI values (minimum of 17 and maximum of 37 kg/m^2^). Although it could be argued that the inclusion of patients with a lower (≤18.5 kg/m^2^: underweight) and higher ( ≥ 30 kg/m^2^: obese) BMI introduced a bias because reference values should be derived from apparently healthy individuals, the main aim of our study was to achieve maximum representativeness from community-dwelling people. There is no consensus in the literature regarding the inclusion of participants with the lowest and highest BMI values for the determination of reference equations for field test where these participants had been excluded from some studies [32,35,38] but not from others [33,43].

The absence of differences between the number of steps achieved by participants and those predicted by the equation in 20% of participants of our sample is considered a strength in our study, suggesting that this equation is valid and can be applied in clinical practice. Another important strength was the accomplishment of the sample size calculated prior to the study, despite the restrictions due to the COVID-19 pandemic during data collection. Additionally, this equation was developed using only age and sex variables, facilitating its direct translation to clinical practice. However, the inclusion of other variables, such as peripheral muscle strength, to explore their influence in the performance of the IST is important in future studies. Additionally, in future studies, a larger sample size is important to determine normative values for the IST, contributing to greater information on its clinical interpretability. 

This study has limitations that are important to mention. Firstly, the use of a convenience sample might have affected the results. More efforts are necessary to recruit participants from different settings and geographical locations to obtain a representative sample because our data collection was only performed in the north of Portugal. Despite the results from this study being obtained from participants over a wide range of between 19 and 93 years of age, it is important to mention that the number of participants in each age decade was not proportional, in which a lower number of participants were observed in the older decades. More participants in older decades are equally important, especially for the determination of normative values.

This is the first study to develop a reference equation for the IST in the Portuguese adult population and, to the best of our knowledge, one of the only studies to determine a national reference equation based on the performance of a step test (number steps). In fact, in the literature, most of the reference equations for step tests are developed with the intent to predict cardiorespiratory fitness, based on the estimation of maximum oxygen uptake [1,44,45,46]. Equations based on the performance of field tests provide advantages in clinical practice, yielding the utility of these tests as an outcome measure of exercise capacity. In addition, they provide an easy interpretation of patients’ exercise capacity and prognosis in different conditions/diseases.

## 5. Conclusions

The established reference equation for the IST demonstrated that age and sex were the determinant variables for the variability of the results. This study also demonstrated that there were no differences between the actual values performed by participants and those obtained by the equation. These results will help to detect people with a lower exercise capacity, yielding the development of exercise programs and the assessment of their effectiveness.

## Figures and Tables

**Table 1:** Characteristics of participants.

Characteristics	Total Sample (*n* = 155)	Reference Equation Sample (80%, *n* = 124)	Validity Sample (20%, *n* = 31)
Age, years (min; max)	47.8 ± 19.7 (19; 93)	48.3 ± 19.8 (19; 93)	51.1 ± 21.4 (22; 89)
Sex, female, *n* (%)	94 (60.6)	75 (60.5)	18 (58.1)
Height, m	1.66 ± 0.1	1.66 ± 0.1	1.67 ± 0.1
Weight, kg	68.7 ± 13.2	68.1 ± 13.0	71.0 ± 11.5
BMI, kg/m^2^ (min; max)	24.9 ± 3.6 (17; 37)	24.6 ± 3.6 (17; 37)	25.4 ± 3.5 (20; 32)
Smoking status, *n* (%)			
Current smokers	31 (20.0)	22 (17.7)	6 (19.4)
Past smokers	25 (16.1)	23 (18.5)	5 (16.1)
Never smokers	98 (63.2)	78 (62.9)	20 (64.5)
Physical activity (score 0–8)	3 [1–4]	2 [1–4]	2 [1–4]
Physical activity category, *n* (%)			
Insufficiently active	85 (54.8)	69 (55.6)	19 (61.3)
Sufficiently active	70 (45.2)	55 (44.4)	12 (38,7)

Data are expressed as mean ± standard deviation, absolute frequency (%), or median [interquartile range], unless otherwise stated. Legend: min, minimum; max, maximum; BMI, body mass index.

**Table 2:** Median values of performances on the incremental step test by age decade.

	Age Groups (Years)						
Total (*n* = 155)	18–29 (*n* = 33)	30–39 (*n* = 27)	40–49 (*n* = 25)	50–59 (*n* = 29)	60–69 (*n* = 10)	70–79 (*n* = 19)	≥80 (*n* = 12)
IST, number steps	360 [360,360] ^a^	360 [329–360] ^a^	303 [272–360] ^b^	300 [235–320] ^b^	183 [118–288] ^c^	97 [21–137] ^d^	50 [13–61] ^e^

Data are expressed as median [interquartile range]. Legend: IST, incremental step test. ^a^ Different from 60–69, 70–79 and ≥80 years. ^b^ Different from 70–79 and ≥80 years. ^c^ Different from 18–29 and 30–39 years. ^d^ Different from all age groups, except 60–69 and ≥80 years. ^e^ Different from all age groups, except 60–69 and 70–79 years.

**Table 3:** Multiple linear regression analysis with the incremental step test.

		Unstandardized Coefficients		Standardized Coefficients			
	r^2^	B	SE	β	95% CI	*p* value	SE of estimate
IST	0.60						
Constant		475.52	19.0		437.9 to 513.1		77.9
Age		−4.68	0.35	−0.76	−5.4 to −4.0	<0.001	
Sex		30.50	14.20	0.12	2.39 to 58.62	0.03	

Legend; IST, incremental step test; B, unstandardized coefficients; β, beta (standardized coefficient); CI, confidence interval; SE, standard error.

## Data Availability

The data are available upon request from the corresponding author.
